# Ultrasonic Navigation for the Treatment of Medication-Related Jaw Osteonecrosis Involving the Inferior Alveolar Nerve: A Case Report and Protocol Review

**DOI:** 10.3390/mps3040070

**Published:** 2020-10-21

**Authors:** Gerardo Pellegrino, Francesca Pavanelli, Agnese Ferri, Giuseppe Lizio, Roberto Parrulli, Claudio Marchetti

**Affiliations:** Oral of Maxillofacial Surgery Unit, DIBINEM, University of Bologna, Via San Vitale 59, 40125 Bologna, Italy; francesca.pavanelli@studio.unibo.it (F.P.); agnese.ferri3@unibo.it (A.F.); giuseppe.lizio@libero.it (G.L.); roberto.parrulli2@unibo.it (R.P.); claudio.marchetti@unibo.it (C.M.)

**Keywords:** MRONJ, dynamic navigation, ultrasonic instrumentation

## Abstract

Dynamic navigation (DN) is a computer-guided technique employed in different surgical fields and recently adopted in dental implantology to improve the accuracy of dental implant insertion. Medication-related osteonecrosis of the jaws (MRONJ) often requires the surgical removal of the impaired, hard tissue, trying at the same time to spare the healthy tissue and the noble anatomical structures. A case of extensive bilateral medication-related osteonecrosis, with the symptomatic involvement of the right mandibular canal, was successfully resolved with the use of ultrasonic surgery associated with a dynamic navigation, in order to limit the invasiveness of the surgical approach improving its reliability and accuracy. The usefulness of this technology in the management of MRONJ can be considered in future clinical trials to confirm the advantages and standardize the technique.

## 1. Introduction

Medication-related osteonecrosis of the jaws (MRONJ) remains a controversial pathology, due to a not completely clear etiology [1]. To achieve the illness resolution, attested by a complete bone tissue covering by the mucosa without any symptoms, different approaches have been discussed according to the stage of the disease, the entity of exposition and the patient’s symptoms [1,2,3,4]. The complete removal of the necrotic bone during surgery is a key point for healing achievement [2], and ultrasonic surgery was revealed to be quite useful in carefully removing the impaired tissue with respect of the nerves and vessels: the micro vibrations generated by this instrumentation, unlike a conventional drill system, obtain a linear and secure cut of the bone, maintaining its vitality and structure [5,6,7].

Dynamic navigation (DN) is a computer-guided technology that allows the surgeon to track the position of the surgical instruments onto a screen displaying the cone-beam CT images in real-time. It works thanks to a camera recording the position of reference tools placed on the patient and on the operating handle piece and to a software able to match these data with CT ones, which were pre-operatively acquired. Several surgical fields take advantage of DN technology, from neurosurgery to maxillofacial surgery, aiming to improve accuracy and to reduce the invasiveness and complications [8]. As this technology became more and more manageable as time went by, it was employed successfully in a smaller surgical field. Recently, thanks to the intraoral application of the patient reference tool, differently from the bulky cranial bone anchorage in maxillofacial surgery, some navigation systems have been employed in dental implantology. The technological support for this field of dentistry obtained better results in terms of implant positioning accuracy than with a traditional free-hand approach [9,10,11].

The combination of the surgical ultrasonic instruments with DN, defined as “ultrasonic navigation”, further improved the quality of implantology, reducing the training time of the novices and allowing a flapless approach with respect to the bone profile and architecture [12].

The present paper aims to report about the successful use of “ultrasonic navigation” in a case of extended mandibular osteonecrosis involving the inferior alveolar nerve in terms of clinical and radiological results.

## 2. Experimental Design

### 2.1. Case Presentation

A 75-year-old man was referred in July 2019 to the Oral and Maxillofacial Department of Bologna (University of Bologna, Italy) for the management of a suspected MRONJ. In 2013, he underwent total thyroidectomy and metabolic radiotherapy with iodine-131 for a thyroid follicular carcinoma. As bone metastases appeared in the left iliac wing, the patient underwent a radiotherapy cycle on this site. In January 2014, he started a monthly therapy with zoledronate 4 mg (43 administrations) that was suspended in July 2017 to allow dental extractions. In February 2016, he started a daily oral therapy with sorafenib which was suspended because of severe stomatitis. From June to December 2016, he took lenvatinib 20 g per os per day and a reduced dosage of 10 mg from January 2017. He was also on therapy with levothyroxine sodium, candesartan cilexetil, lacidipine, and pantoprazole.

Both first inferior molars were extracted in 2017 and the postoperative healing of the extraction sites was incomplete. In the subsequent two years, the patient suffered from recurrent abscesses managed with multiple cycles of antibiotic therapy.

**Figure 1:**
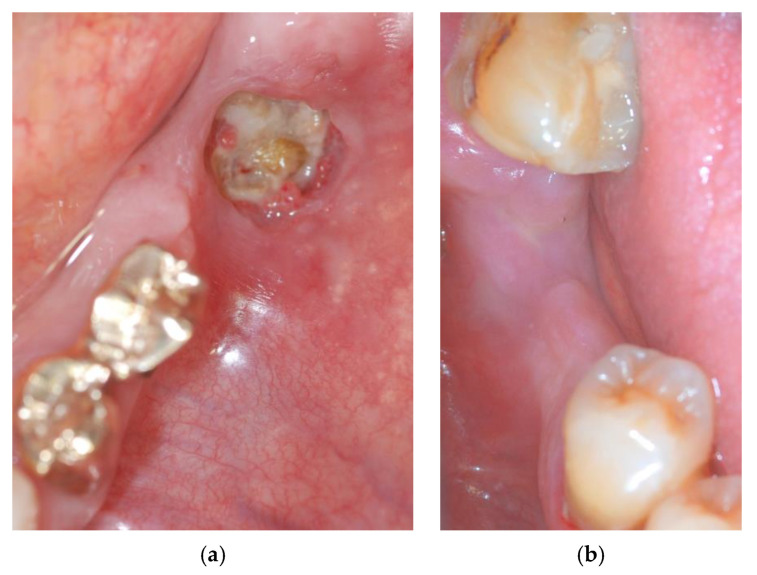
The exposed necrotic bone in the left lower edentulous first molar region before the surgery (**a**); the region affected by medication-related osteonecrosis of the jaws (MRONJ) on the right side before the surgery (**b**).

Clinical examination showed exposed necrotic bone and the presence of purulent exudate on the left unhealed site (Figure 1a) and fistula with purulent drainage on the right side (Figure 1b).

**Figure 2:**
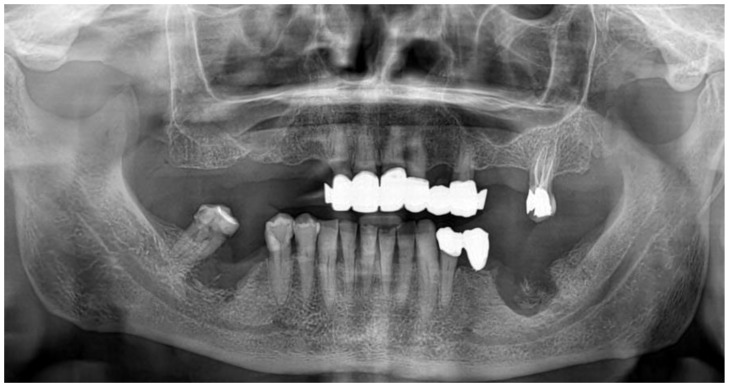
The panoramic radiograph showing the two regions affected by MRONJ.

The patient complained of spontaneous pain and paresthesia in the lower right lip. The evaluation of the panoramic radiograph highlighted the presence of altered bone morphology with osteolytic and osteosclerotic areas in both sites, the involvement of the right-sided mandibular canal and the presence of a bony sequestrum on the left side. (Figure 2).

Clinical and radiographic signs oriented for a diagnosis of stage 2 (AAOMS staging) [13] bilateral MRONJ.

**Figure 3:**
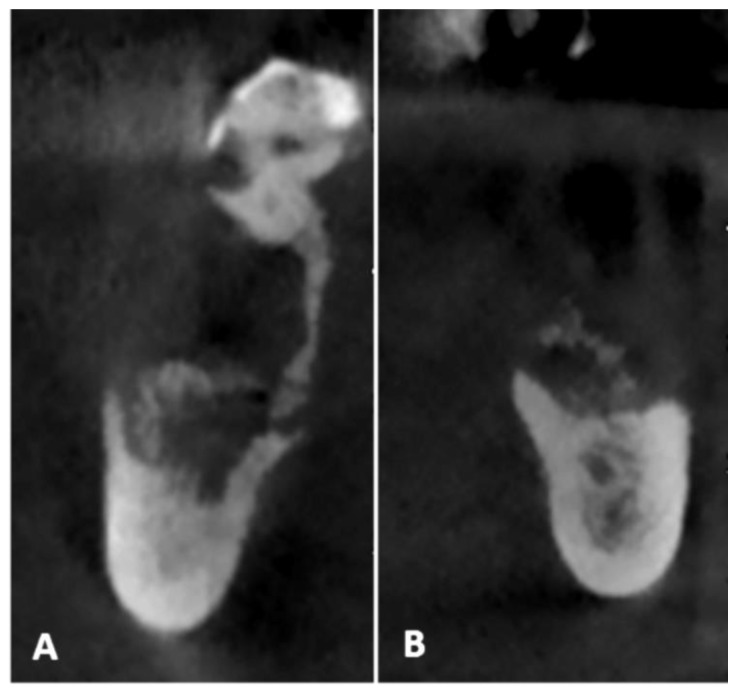
The presence of altered bone morphology on the preoperative CBCT (**A**,**B**), with the involvement of the right-sided mandibular canal with loss of root continuity (**A**).

Considering the extension of the osteonecrotic areas and the partial involvement of the right mandibular canal, a surgical curettage was planned under local anesthesia with the support of ultrasonic navigation. All the procedures followed the ethical standards of the responsible committee on human experimentation and the Helsinki Declaration. ImplaNav (BresMedical, Sydney, Australia) navigation system was used in both the planning and surgical phases. The patient underwent a cone-beam CT with a reference tool fixed on his inferior teeth. The CBCT (cone-beam computed tomography) images confirmed the involvement of the right-sided mandibular canal with loss of roof continuity (Figure 3A) and the presence of a bone sequestrum on the left side (Figure 3B).

The patient took antibiotic prophylaxis, starting three days before surgery, with amoxicillin + clavulanic acid (1 g/8 h) and metronidazole (250 mg/8 h) and 0.2% chlorhexidine digluconate mouth rinses. No drug holiday was requested.

Before the surgery started, the reference tool was worn by the patient and the tracking arrays were positioned on it (patient reference tool) and on the piezoelectric handpiece (handle reference tool). Then the calibration of the system was made, allowing the infrared stereoscopic camera to record the patient and ultrasonic tip position.

**Figure 4:**
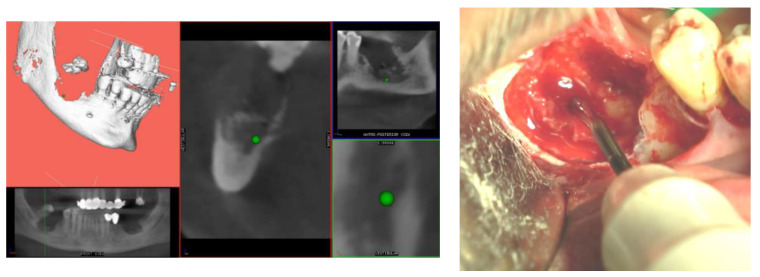
The navigated piezoelectric bone curettage on the right side.

After the patient signed the intervention consent form, local anesthesia was performed. A full-thickness flap was elevated on both sides, the bone sequestrum was removed and bone curettage was performed with ultrasonic tips (Esacrom, Imola, Italy) and using the navigation system. Thanks to this guided technique, the surgeon was able to follow in real-time the ultrasonic tip position on the system screen displaying the CBCT images, and safely identify and remove the necrotic bone surrounding the inferior alveolar nerve on the right side (Figure 4).

The bone curettage was carried out until a bleeding bone was reached. Then the flaps were released with periosteum cutting and tightly sutured. Antibiotic assumption was prolonged for 7 days thereafter, along with ibuprofen 600 mg as needed, arnica composite, 15 drops three times a day, and alpha lipoic acid 800 mg a day for 10 days.

**Figure 5:**
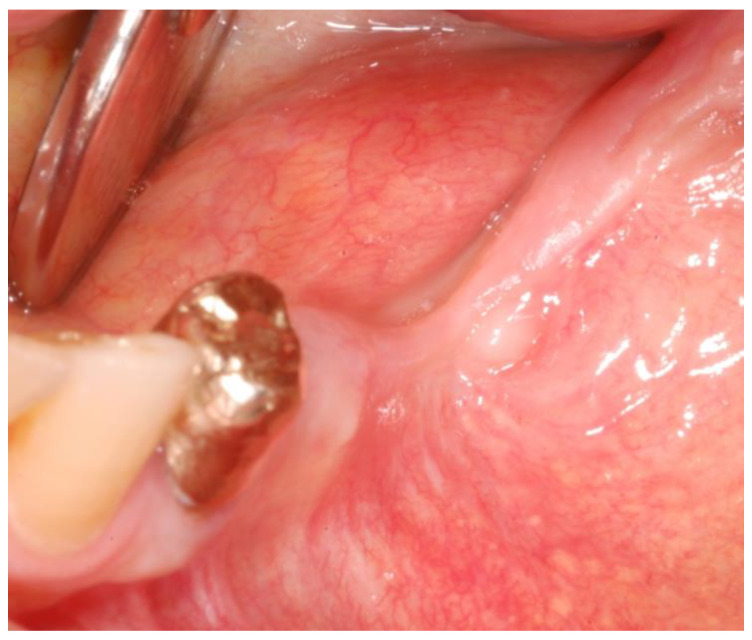
The 3-month postoperative healing of the soft tissue without bone exposure on the left side.

**Figure 6:**
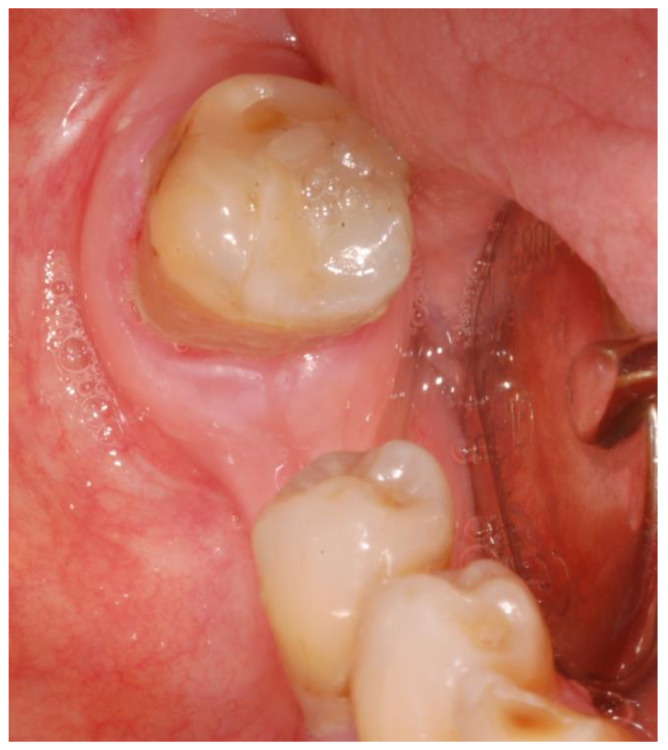
The 3-month postoperative healing on the left side.

**Figure 7:**
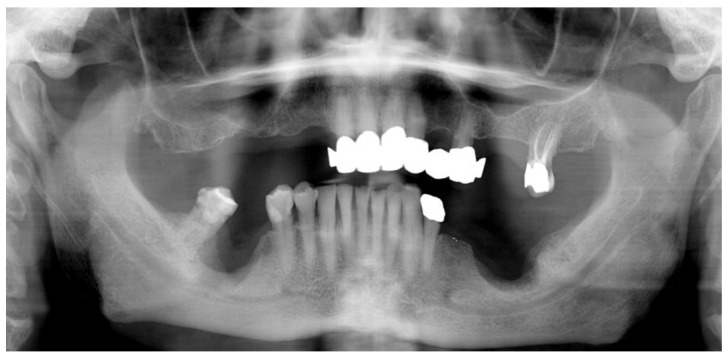
The 3-month postoperative panoramic radiograph.

Complete soft tissue healing was recorded two weeks after surgery, with no postoperative pain and a significant reduction in lip para-anesthesia. The 3-months postoperative follow-up visit, and the radiographic evaluation revealed successful healing without bone exposure and no signs of inflammation (Figure 5, Figure 6 and Figure 7).

### 2.2. Materials

- ImplaNav (BresMedical, Sydney, Australia) navigation system.
- Esacrom, (Imola, Italy) piezo-surgical instruments.

## 3. Procedure

1. Pre-operative evaluation. The patient undergoes a cone-beam CT exam with a reference plate containing the fiducial markers, fixed on his inferior teeth. The Digital Imaging and Communications in Medicine (DICOM) were imported into the navigation software.
2. Just before surgery, the patient wears the reference plate with the patient reference tool attached onto it and the handle reference tool on the piezoelectric handpiece. The calibration of the navigator system, accomplished in this phase, is performed through a connection reproducing the universal joint for the drills embedded into the calibration tool, allowing the navigation system software to identify the drill position and axis in relation to the patient position and to the CT imaging data.
3. Surgery timing. The surgeon can follow in real time the ultrasonic tip position onto the system screen displaying the CBCT images and safely identify and remove the necrotic bone surrounding the inferior alveolar nerve on the right side.

## 4. Discussion

The key points for achieving post-operative healing of MRONJs are the complete removal of the infected necrotic bone and the preservation of soft tissues and delicate anatomical structures [1,2].

Since to date there is not a diagnostic technology able to reliably distinguish the ill bone from the healthy one, the possibility to be surgically radical has been questioned, even in consideration of the biological costs. In any case, it is still debatable as to when a conservative approach is required, with only a removal of spontaneous sequestrum under antibiotic therapy and topic disinfection, or a surgical intervention with the raising of extended flaps and further bone tissue exposure to the oral environment [1,2,3,4]. Actually, after a first conservative choice, when the symptoms persist, the surgical removal of the pathologic hard tissue seems to be unavoidable and the risk of dramatic incidents and complications must be taken into consideration [1].

The first factor influencing the treatment strategy is the entity of local involvement of the jaw bones: if osteonecrosis is confined above the mandibular canal and below the maxillary sinus, sequestrectomy and/or bone curettage under local anesthesia can be resolutive; alternatively, it is necessary a complete bone resection immediately followed by reconstruction with fibula or scapula free flaps, even for the prevention of future possible fractures of the residual bone [10,11].

The second factor to be considered in the intervention is the general medical situation of the patients related to their primary pathology. The reported case presented a further criticism due to its complex medical therapy: at the time of tooth extraction, the patient was in therapy not only with zoledronate but also with lenvatinib, which is a multikinase inhibitor with antiangiogenic action. Even though European Pharmacovigilance Agencies still do not include osteonecrosis among lenvatinib’s side effects, Mauceri et al. [14] reported the occurrence of a post-extraction osteonecrotic lesion correlated with the assumption of this drug.

The therapeutic decision can depend, moreover, on the technical possibilities and the operator’s skills and experience, particularly treating out-patients in local anesthesia.

Ultrasonic navigation helped to successfully address several problems implied in the patient situation here reported. First of all, the real-time monitoring on the three dimensional radiological anatomy of the position of ultrasonic instruments is simpler to control than the rotational one, which allows the operator to better manage the bone removal according to the chromatic distinction between the sclerotic bone and the normal bone. Continuously comparing this information with the macroscopic features of the tissues, just like the bleeding for the healthy bone, can improve the radicality of the intervention. Along with this, the regular and vital edges of bone cuts obtained with ultrasonic instruments, unlike the necrotized and jagged ones by drilling systems, should be preferred, particularly in treating bone affected by medicine-related osteonecrosis [5,7]. Blus et al. reported the complete healing of MRONJ in twenty patients treated with ultrasonic surgery: apart from the absence of traumatism and heating, they assumed that the ultrasonic instrumentation can empower the antibiotic effect due to the killing of bacteria in suspension [15].

The ultrasonic navigation enabled us to reach this objective, maintaining the integrity of the periosteum and the oral mucosa to provide the resected bone tissue with a vital cover during the healing period, avoiding dehiscence and bone exposure. A more accurate extension of bone curettage with a consequent lower surgical trauma and a reduction in surgical time favored less discomfort for the patient and a better post-operative course.

Similarly, the integrity of the alveolar inferior nerve, symptomatically involved in the pathologic process with an interruption of the mandibular canal roof, was preserved in this case with a complete resolution of the sensitive alterations.

No studies on the use of dynamic navigation for the surgical treatment of MRONJ have been published yet. Several model-based ex-vivo and clinical studies demonstrate good accuracy values of the tested navigation systems, higher than those obtained with a conventional free-hand dental implant placement technique [9]. That is because of the possibility to guide every phase in real-time, from the site preparation for the implant placement, relying on both the clinical view and the CBCT images at the same time [8].

In the present case, the dynamic navigation system allowed the virtual 3D planning of the surgery and facilitated the bone curettage in hidden areas, preserving the integrity of the inferior alveolar nerve, thanks to the possibility to detect the tip position of the CBCT images. The procedure was accomplished by a medium-level operator in a dental office.

Although further studies are required to investigate the MRONJ treatment, this first pilot case showed encouraging results and could suggest a reliable and safe surgical approach.
